# Inequities and factors associated with gender and disability in NTD-endemic communities in Benin and Côte d'Ivoire: an intersectional approach to interventions

**DOI:** 10.3389/fgwh.2025.1575082

**Published:** 2025-04-08

**Authors:** Ginette Victoire Senami Gandigbe, Zinsou Franck Mignanwande, Yévèdo Borel Tossou, Karine Lucrèce Marie Codjo-Seignon, Sonagnon Inès Elvire Agbo, Fifamin Noël Christelle Gbaguidi, Sèdjro Gimatal Esaï Anagonou, Fernand Aimé Guedou, Mark Nichter, Cristina Juan Jimenez, Roch Christian Johnson, Anna Gine-March

**Affiliations:** ^1^ Ecole Doctorale des Sciences de la Santé de Cotonou, Université d’Abomey-Calavi, Cotonou, Benin; ^2^ Centre Interfacultaire de Formation et de Recherche en Environnement Pour le Développement Durable, Université d’Abomey-Calavi, Abomey-Calavi, Benin; ^3^ School of Anthropology, University of Arizona, Tucson, AZ, United States; ^4^ Anesvad Foundation, Bilbao, Spain; ^5^ Fondation Raoul Follereau, Paris, France

**Keywords:** gender, disability, health inequities/health disparities, neglected tropical diseases, Benin, Côte d'Ivoire

## Abstract

**Introduction:**

Gender inequity among people with disabilities (PWD) is a complex issue influenced by a combination of socioeconomic, cultural, political, and institutional factors. Women with disabilities often experience double discrimination, increasing their vulnerability. These exacerbate their already difficult health and living conditions. This study aimed to explore the factors associated with gender inequities among PWD in Benin and Côte d'Ivoire, and to design and implement tailored interventions to address these inequities.

**Method:**

This intervention-oriented operational research, employing a mixed-methods approach (quantitative and qualitative), targeted 841 PWD and 90 community members. Initially, records from various centers managing Neglected Tropical Diseases (NTDs) in the study areas were reviewed. All PWD listed in the registers and residing in the community, as well as other community members, were included. Data on socio-demographic and socioeconomic and clinical factors were collected using an interview guide and an observation grid. Quantitative data were analyzed using SPSS version 25 (alpha = 5%), while qualitative data were processed through triangulation, categorized, cross-tabulated, and synthesized by theme, hypothesis, and indicator. A subset of PWD facing barriers to accessing education, training, income-generating activities, or healthcare was selected for intervention.

**Results:**

The median age was 38 years (IQR = 22–52). Among the 841 PWD, 497 were men and 344 were women (sex ratio = 1.45). Univariate analysis revealed significant associations (*p* = 0.000) between gender disparities and marital status, monthly income, education level and occupation type. Multivariable analysis identified three factors strongly associated with gender disparities (*p* = 0.000): marital status, monthly income, and profession. Interventions were designed to address these factors and included vocational training, medical care, and educational or professional scholarships. These measures collectively improved the living conditions of PWD and helped raise awareness among those in their immediate environment, fostering social integration and reducing self-stigmatization.

**Conclusion:**

This study provides a deeper understanding of the inequalities that PWDs face in Benin and Côte d’Ivoire. It highlights the need to adopt intersectional approaches in policies and practices to effectively address the multiple forms of discrimination faced by women and girls with disabilities, promoting their inclusion and protection in all areas of society.

## 1 Introduction

Health inequalities, gender, and disability intersect in complex ways, with people living with disabilities facing increased vulnerability due to both their disability and gender. Women and girls with disabilities often experience multiple layers of discrimination, including barriers to healthcare access, education, and employment opportunities (1, 2). The socioeconomic and structural factors limiting healthcare access for women with disabilities globally (1) and health disparities exist between women with and without disabilities, covering chronic diseases, mental health, health promotion behaviors, and healthcare utilization (2).

Disability is a global public health issue. According to the United Nations, by 2023, more than a billion people, or approximately 15% of the world's population, will be affected by disability (3, 4). This figure is constantly increasing, notably owing to the aging of the population and the increasing prevalence of noncommunicable diseases (3) and neglected tropical diseases with cutaneous manifestations (NTDs-CM). According to the International Classification of Functioning, Disability, and Health (ICF), disability comprises several components including health problems, organic functions and anatomical structures, activities, participation, and personal and environmental factors (5). However, in physical terms, it is most often an individual characteristic, resulting directly from the alteration of an organ: this is the impairment (3). This impairment can lead to functional limitations, depending on the part of the body affected. NTDs such as leprosy and Buruli ulcer can lead to disabilities such as visual and physical impairments but also mental health problems, stigma, and discrimination (6, 7). Skin-related Neglected Tropical Disease (SNTDs) are particularly stigmatizing due to their visible nature, rendering affected individuals vulnerable to psychosocial risk and the associated decline in social participation, quality of life and mental health (8–10). Men and women with NTDs have reported debilitating physical symptoms that have hampered their ability to work, socialize and carry out their usual daily activities. Many men and most women were stigmatized, discriminated by others, for fear of infection. These behaviors had consequences for mental well-being such as mood lows, anxiety, low self-esteem and suicidal thoughts. Disease-specific knowledge, early treatment, social support and acceptance of the disease were mentioned as protective factors (8).

Therefore, the integration and well-being of PWD are crucial issues for society, particularly in developing countries such as Benin and Côte d'Ivoire (11). The term “people with disability (PWD)” includes all people with one or more disabilities (motor, sensory, cerebral) regardless of the cause (including NTDs). NTDs perpetuate poverty, while access to healthcare, education, and income generating activities are crucial for achieving acceptable living conditions (12).

Studies have shown that women and girls with disabilities face significant discrimination in terms of access to healthcare, education and employment (13). The health of women and men is different and unequal: “Sex differences in health are usually described as a paradox, where women have longer life expectancy than men but poorer health status, in terms of indicators such as mental health, chronic illness, disability, or self-rated general health” (14–16).

Equity in health is a fundamental human right, ensuring that all individuals, regardless of race, religion, political belief, economic or social condition, including gender, have the opportunity to reach the highest attainable standard of health. Health inequities are preventable, unfair differences in health risks, outcomes, and their social and economic consequences. These inequities reflect moral and ethical concerns, as they involve avoidable and unjust disparities between groups. Unlike inequalities, which refer to measurable differences, inequities point to differences that are not only avoidable but also unjust and discriminatory (17).

In healthcare, access to care is unequal for men and women (18). Disparities in health outcomes cannot be explained solely by genetic or physiological differences between the sexes, but are also the result of inequalities in opportunities to enjoy good health. Gender intersects with other social positions to hamper access to healthcare like socioeconomic, geographic, and cultural factors and create structural barriers when accessing healthcare. According to the World Health Organization, PWD have unequal access to healthcare services. Indeed, gender norms and power relations influence women's access to health services and timely diagnosis (18).

PWD also face socioeconomic, cultural, or social challenges, compounded by gender inequalities. The intersection of disability and gender has been the subject of various studies that highlight how women and girls with disabilities face multiple and simultaneous forms of discrimination, exacerbating their vulnerability in different areas. Indeed, women and men with disabilities can experience very different realities due to gender roles, social expectations, and gender-specific discrimination, which are exacerbated among PWD (19).

According to The European Institute of Gender Equality, women with disabilities are at a disadvantage in the labor market, face significant barriers to accessing education (19, 20). In Africa, according to the United Nations, the human development index is higher for men than for women, and men have higher average incomes and higher levels of education than women do (21). According to the same institution, certain factors contribute to the gap between men and women in terms of disability. These factors include the impact of the invisibility of women and girls with disabilities in work on women, disability rights and development; the double discrimination faced by women and girls with disabilities, which is often exacerbated by other factors such as being a minority, an Indigenous person, a refugee, a person living with human immunodeficiency virus (HIV) and acquired immunodeficiency syndrome (AIDS) or elderly; and the lack of empowerment and capacity development among women and girls with disabilities, particularly in terms of leadership and participation in decision-making in the political, economic and social spheres (22).

In Africa, and more specifically in Benin and Côte d'Ivoire, very little work has been devoted to studying the factors associated with gender disparities and inequities among PWD. In 2019, a study conducted in Benin titled “The gendered impact of Buruli ulcer on the household production of health and social support networks “concluded that” while men are the primary decision makers for healthcare decisions outside the home, women are largely responsible for arranging care for the afflicted in hospital in addition to managing their households” (23). Another study concluded that gender inequities in the school environment are legitimized by the social roles assigned distinctly to girls and boys with the school institutions helping to perpetuate them (24). An additional study showed that disabled women feel overwhelmingly rejected by their relatives. This creates within them feelings of inferiority and frustration that sometimes plunge them into isolation and mental depression (25).

This limited number of studies means that there is a real lack of knowledge about the lifestyles of disabled people, the realities they face, and the gender inequities they are confronted with. This lack of information has consequences both for the assessment of impacts and the provision of care adapted to the context and the individual. This study was conducted to fill this gap by identifying the factors. Associated with gender disparities and inequities among PWD and to propose interventions tailored to their living context.

## 2 Study settings and methods

### 2.1 Study settings

Data were collected in the following locations in Benin: Ouidah, Lalo, Allada, and Pobè, and in Côte d'Ivoire in Divo, Yamoussoukro and Tiassalé. These localities were selected due to their endemicity for NTDs and the presence of health centers specializing in their management.

### 2.2 Study design

This was an intervention-oriented operational research study employing a mixed-methods approach (quantitative and qualitative), carried out in three stages:

• Mixed methods analysis of factors associated with gender disparities and inequities in PWD
• Design and implementation of appropriate interventions, including medical care, education and training, and income-generating activities
• Evaluation of intervention results

Data was collected in Benin and Côte d'Ivoire from 2022 to 2024.

### 2.3 Target populations

This study included participants with disabilities caused by NTDs or other factors, as well as community members such as families, opinion leaders, and caregivers.

#### 2.3.1 PWD

The registers of the care centers mentioned above were examined. All people with disabilities due to NTDs received at these centers in the last five years were included (2015–2020). The localities of origin were rigorously recorded. The team of researchers then traveled to the villages to locate these people and interview them. In the localities visited, other PWD who were present during the survey and provided consent were also systematically included.

#### 2.3.2 Community members

Family members (ascendants or legal tutors) who provided consent were included. The same applied to people responsible for the care of PWD at various levels (social workers, nurses, doctors, physiotherapists), as well as opinion leaders (village chiefs, religious authorities, tradithérapeutes, etc.).

### 2.4 Sample size

The study population consist of 841 PWD (418 in Benin and 421 in Côte d'Ivoire) and 90 community members (57 in Benin and 33 in Côte d'Ivoire). For the qualitative part of the survey, we interviewed:

• 51 family members, including 35 in Benin and 16 in Côte d'Ivoire.
• 29 care staff members, including 18 in Benin and 11 in Côte d'Ivoire.
• 10 opinion leaders (local elected officials, district chiefs, village chiefs, arrondissement chiefs), including 4 in Benin and 6 in Côte d'Ivoire.

### 2.5 Variables

To identify factors associated with measurable differences between female and male individuals with disabilities (PWD), the following variables were measured:

• **Sociodemographic data:** age, education level, marital status and occupation type;
• **Social data:** restriction of social participation (RSP)
• **Socioeconomic data:** monthly income;
• **Clinical data:** type and cause of disability.

The intervention component was based on the following variables: access to education (at school level); access to training (to achieve professional training), access to an income-generating activity, and access to healthcare.

### 2.6 Data collection tools

Data were collected through structured questionnaires administered to participants with disabilities, community members such as families, opinion leaders, and caregivers.

The questionnaires for PWD included questions on socio-demographic characteristics, social data, socioeconomic data, and clinical data.

For community members and key contact persons, an interview guide was developed and pre-tested for in-depth interviews.

A grid for observing interactions between PWD and their community was also employed to assess these people in their social context. To capture the experiences and interrelations of PWD with their families and communities, the interviewers spent 24− to 48 h with the individuals selected for this in-depth study.

### 2.7 Data processing and analysis

Using the KoboCollect tool, quantitative data was gathered from 841 people with disabilities (PWD). This data was then exported to Excel to ensure its completeness and accuracy. Data analysis was carried out via the Statistical Package for Social Science (SPSS version 25). Quantitative variables are expressed as the means with standard deviations, whereas qualitative variables are summarized as frequencies. Means were compared with Student's *t*-test, and frequencies were compared with Pearson's chi-squared test or Fisher's exact test, as appropriate. A bivariate analysis using logistic regression was performed, and variables with a *p*-value of less than 20% were retained for multivariable analysis. The multivariable analysis was performed using a stepwise iterative logistic regression model (Wald top-down). For all comparisons, a *p*-value of less than 5% was considered to indicate statistical significance.

For qualitative data, interviews with target groups were recorded using a tape recorder. The content of the interview recordings was transcribed in French and coded. This enabled an exhaustive, interpretative description of the flow of the interviewees' discourse with a procedure for saving it “in legible terms” (26). This input procedure allowed the data to be categorized, cross-referenced, and synthesized thematically according to previously defined hypotheses and indicators. Based on the triangulation of the different points of view that emerged from the interviews, the data analysis was structured around the main lines retained after analysis of the content of the interviews, the results, and the major questions to be answered by the study.

### 2.8 Intervention

Following data collection and analysis, interventions were implemented to improve the quality of life of PWD and reducing gender inequities. These interventions consisted of:

• Medical care

Medical care for PWD, including diagnosis, treatment, rehabilitation, and follow-up.

• Education and training

Support for PWD in their schooling and professional training.

• Income-generating activities

The aim was to put in place measures allowing PWD to have their own source of income to improve their living conditions.

### 2.9 Intervention result assessment

The postintervention results were assessed in three steps:

• Individual level

These changes were due to the intervention and observed at the individual level; examples include a reduction in self-stigmatization or self-fulfillment of the PWD.

• Household- level

These changes were observed at the household level; examples include increased household income and improved living conditions (access to water, hygiene and sanitation, food, and health care).

• Community level

These changes were observable in society; examples include the participation of people with disabilities in community life and the integration of PWD into groups or unions.

### 2.10 Ethical aspects

The study was approved by the research ethics committee of the University of Parakou (CLERB-UP) in Benin (authorization No. 0492/CLERB-UP/P/SP/R/SA of 19/11/2021) and by the National Ethics Committee for Life Sciences and Health (CNESVS) of Côte d'Ivoire (authorization N/Réf: 076-22/MSHPCMU/CNESVS-kp of 28/07/2022). All participants provided written informed consent after the research team explained the study's objectives and ensured they understood participation was voluntary and refusal had no consequences and Written informed consent was obtained from the [individual(s) AND/OR minor(s)' legal guardian/next of kin] for the publication of any potentially identifiable images or data included in this article.

## 3 Results

### 3.1 Quantitative analysis

#### 3.1.1 Characteristics of people with disabilities and community members

The survey was carried out on 841 people with disabilities (PWD), including 418 (49.7%) in Benin and 423 (50.3%) in Côte d'Ivoire.

Participants' ages varied widely, from 1 to 115 years. The median age was 38, with the middle 50% of ages falling between 22 and 52. Three-quarters of the respondents were aged between 15 and 65 years.

Among the PWD, 497 (59.1%) were men, and 344 (40.9%) were women (sex ratio = 1.45)

The analyses of school enrollment were limited to PWD who were at least 5 years of age (school enrollment age). Of the 828 participants who met this criterion, 295 (35.6%) had attended school: 158 (19.0%) at the primary level, 111 (13.4%) at the secondary level, and 26 (3.14%) at the tertiary level. The remaining 533 or 64.4% of participants were not currently in school [305 (61.2%) men and 228 (67.7%) women].

The analysis of marital status and occupation type was limited to the 733 eligible participants (aged 15 years and older). Among this subpopulation, married participants predominated (43.7%), followed by single participants (35.6%).

In terms of profession, 535 participants (73.1%) had no profession, and 198 (27.0%) reported having a profession; among the latter, 77 (38.9%) were craftsmen/workers, 47 (23.7%) were farmers/peasants, and 17 (08.6%) were civil servants.

About monthly income, the majority of respondents (89.2%, or 654 individuals) had very low incomes, ranging from 0 to 76 euros.

With respect to the number of disabilities, 772 respondents (91.8%) had a single disability, whereas the remaining 69 (08.2%) had multiple disabilities. A total of 554 PWD (65.8%) had an isolated motor disability. Among the PWD, 173 individuals (20.6%) presented with an isolated sensory disability (visual, auditory, olfactory, or tactile). Isolated cerebral palsy (CP) was found in 45 (05.4%) of the PWD surveyed.

Only 98 (11.7%) PWD had disabilities due to NTDs.

The majority, i.e., (38) of the interviewed family members were aged 30–59 years; 25 were men, and 26 were women (sex ratio = 1). Of the family members, 25 had received formal education: 12 at the primary level, 12 at the secondary level, and 1 at the tertiary level. The remaining 26 family members had no schooling. Among the family members, 37 were married, 30 were Christians, and 14 practiced their ancestral religion. The majority, i.e., (22) caregivers were aged between 30 and 59 years. Among this population, 14 were men, and 15 were women (sex ratio = 0.9). Almost all, i.e., 22 care professionals were educated at the university level. Married people predominated at 19. The care professionals included 3 doctors, 7 nurses and midwives, 4 physiotherapists, 5 social workers, and 3 traditional healers.

The majority, i.e., (9), of the community leaders were aged 30–59 years; 9 (90.0%) were men, and 1 was a woman (sex ratio=9). Of the community leaders, 9 had received formal education: 1 at the primary level, 4 at the secondary level, and 4 at the tertiary level. Only 1 community leader had not attended school. In terms of marital status, 8 community leaders were married.

#### 3.1.2 Gender disparities and inequities and disabilities

In the univariate analysis (Table 1), there was no association between gender and variables such as age (*p* = 0.324), the RSP (*p* = 0.616), the management of PWD (*p* = 0.747) and the type of disability (*p* = 0.053).

**Table 1:** Bivariate analysis of the association between sex and demographic, economic, and medicosocial characteristics of PWD (with or without NTD-related disabilities) in Benin and Côte d'Ivoire.

Variables	Female sex		OR	95% CI	p*
	No *n* (%)	Yes *n* (%)			
Age (years)	*n* = 497	*n* = 344			0.324
[0–14]	65 (60.2)	43 (39.8)	1	1	
[15–29]	110 (57.0)	83 (43.0)	1.14	[0.71–1.84]	
[30–44]	122 (58.4)	87 (41.6)	1.08	[0.67–1.73]	
[45–59]	125 (62.2)	76 (37.8)	0.92	[0.57–1.48]	
[60–74]	59 (63.4)	34 (36.6)	0.87	[0.49–1.54]	
75 and over	16 (43.2)	21 (56.8)	1.98	[0.93–4.22]	
Marital status	*n* = 432	*n* = 301			0.000
Married/In a couple	214 (66.9)	106 (33.1)	1	1	
Single	165 (63.2)	96 (36.8)	1.17	[0.83–1.65]	
Divorced/Separated	37 (56.1)	29 (43.9)	1.58	[0.92–2.71]	
Widowed	16 (18.6)	70 (81.4)	8.83	[4.89–15.95]	
Monthly income (Euro)	*n* = 432	*n* = 301			0.000
[0–76]	362 (55.4)	292 (44.6)	1	1	
More than 76	70 (88.6)	09 (11.4)	0.16	[0.08–0.32]	
Education level	*n* = 491	*n* = 337			0.000
No schooling	305 (57.2)	228 (42.8)	1	1	
Primary	83 (52.5)	75 (47.5)	1.21	[0.85–1.73]	
Secondary/Tertiary	103 (75.2)	34 (24.8)	0.44	[0.29–0.67]	
Profession	*n* = 432	*n* = 301			0.000
No profession	272 (50.8)	263 (49.2)	1	1	
Civil servant	15 (88.2)	02 (11.8)	0.14	[0.03–0.61]	
Craftsman/Worker	60 (77.9)	17 (22.1)	0.29	[0.17–0.52]	
Shopkeeper/Housekeeper	14 (53.8)	12 (46.2)	0.89	[0.40–1.95]	
Farmer	43 (91.5)	04 (08.5)	0.10	[0.03–0.27]	
Other	28 (90.3)	03 (09.7)	0.11	[0.03–0.37]	
Type of disability	*n* = 497	*n* = 344			0.053
Motor disability	332 (59.9)	222 (40.1)	1	1	
Sensory disability	93 (53.8)	80 (46.2)	1.29	[0.91–1.81]	
CP***	34 (53.8)	11 (24.4)	0.48	[0.24–0.97]	
Multiple disabilities	38 (55.1)	31 (44.9)	1.22	[0.73–2.02]	
RSP****	*n* = 497	*n* = 344			0.616
Yes	324 (58.5)	230 (41.5)	1	1	
No	173 (60.3)	114 (39.7)	0.93	[0.69–1.24]	
Management	*n* = 497	*n* = 344			0.747
Yes	91 (60.3)	60 (39.7)	1	1	
No	406 (58.8)	284 (41.2)	1.06	[0.74–1.52]	

p*, *p*-value; CI, confidence interval; OR**, odds ratio; CP***, cerebral palsy; RPS****, restriction of social participation.

On the other hand, gender was significantly associated with marital status (*p* = 0.000), monthly income (*p* = 0.000), level of education (*p* = 0.000) and occupation type (*p* = 0.000).

In the multivariable analysis (Table 2), three factors that were significantly related to gender were identified: marital status (*p* = 0.000), monthly income (*p* = 0.000), and occupation type (*p* = 0.000).

**Table 2:** Study of the associations between sex and several demographic and economic characteristics among PWD (with or without disabilities due to NTDs) in Benin and Côte d’Ivoire (multivariable analysis).

Variables	Female sex		ORa	95% CI	p*
	No *n* (%)	Yes *n* (%)			
Marital status	*n* = 432	*n* = 301			0.000
Married/In a couple	214 (66.9)	106 (33.1)	1	1	
Single	165 (63.2)	96 (36.8)	0.99	[0.68–1.44]	0.959
Divorced/Separated	37 (56.1)	29 (43.9)	1.30	[0.73–2.33]	0.373
Widowed	16 (18.6)	70 (81.4)	7.32	[3.88–13.82]	0.000
Monthly income (Euro)	*n* = 432	*n* = 301			0.000
[0–76]	362 (55.4)	292 (44.6)	1	1	
More than 76	70 (88.6)	09 (11.4)	0.23	[0.10–0.51]	0.000
Profession	*n* = 432	*n* = 301			0.000
No profession	272 (50.8)	263 (49.2)	1	1	
Civil servant	15 (88.2)	02 (11.8)	0.45	[0.08–2.46]	0.356
Craftsman/Worker	60 (77.9)	17 (22.1)	0.36	[0.20–0.64]	0.001
Shopkeeper/Housekeeper	14 (53.8)	12 (46.2)	1.15	[0.49–2.67]	0.752
Farmer	43 (91.5)	04 (08.5)	0.12	[0.04–0.36]	0.000
Other	28 (90.3)	03 (09.7)	0.16	[0.05–0.54]	0.003

*p*, *p*-value; CI, confident interval; Ora, odds ratio adjusted.

Compared with PWD who were in a union, those who were divorced/separated (or widowed) had a greater probability of being female, and this association was especially significant for the widowed category (*p* = 0.000). On the other hand, the probability of being female was almost the same for single people as for those who were married/in a union.

In terms of occupation, female PWD were less likely to be in occupational categories such as civil servant craftsman/worker (, farmer (and other occupations (musician, volunteer, informal worker) than in the “no profession” category. This association was significant for cultivators, craftsmen, and “other occupations”. On the other hand, female PWD were relatively more likely to be in the Shopkeeper/Housekeeper category than in the “no profession” category However, this association was not significant (*p* = 0.752).

Among people with lower incomes, the probability of being a woman is higher than among people with high incomes

### 3.2 Qualitative analysis

#### 3.2.1 Relationship between marital status and loneliness

The analysis of qualitative data revealed that women with disabilities encounter difficulties in finding a life partner and starting a family, unlike their counterparts without disabilities.

Mrs. A.L., the married mother of one child, is her husband's third wife and is very unfulfilled in her life. Despite being a married mother of one, Mrs. A.L. feels deeply unfulfilled, a situation compounded by her status as her husband's third wife.

“Since the disease made me disabled, my entire family abandoned me, especially on my father's side, after my father died. For a while, they accepted that I would stay with them, but since my disability has worsened, no one will allow me to come to them. However, my mother is experiencing deep depression. Since I got married, only my husband has provided for me.”

Faced with these situations, most husbands adopt a protective attitude, showing courage, experiencing self-denial and using the means at their disposal to ensure their wives' development. These situations often prompt husbands to take on a protective role, which may involve acts of courage, self-denial, and the use of available resources to support their wives.

This was the case for Mrs. M.A.'s husband, who stated the following:

“When I married my wife, she already felt a certain shame and did not like to approach people. She was so reserved. I had to talk to her a lot, and I always show her that she is like other people and that it is because of her disease that she is like that. She and I do the same job, and together, we evolve. I thank the awareness team, especially the Disability Project team, which allowed my wife to find her smile again. Today, my wife and I are doing well. I am a tailor, but because of the problems, I became a motorcycle taxi driver. However, since my wife has her workshop, we work together. During the festive period, we stay up to sew people's clothes. Before you arrived, she was the one who went to people to invite them. I thank God that everything is fine” (husband of M.A., Bonou-Benin).

This interview showed that the husbands of women with disabilities are determined to ensure their wives' well-being and development.

#### 3.2.2 Difficulties in obtaining sufficient income

Most of the women with disabilities did not have enough income to meet their basic needs, even when working. Mrs. A.Y., who has a disability, described her difficulties in the quest for survival income:

“Being a disabled person like me without both feet is not easy. I have to eat, and I don't want to beg. That's why with the help of my family, I was able to create this small business. I sell a few things at home here. I also learned to braid hair while watching others do it. However, again, it's difficult; customers have to sit on the ground so that I can braid their hair. Many don't like it… If I find funding or financial aid, I can buy things and resell them to find money to take care of myself and my child.” (Mrs. A.Y., disabled reseller, Pobè-Benin).

#### 3.2.3 Lack of a profession and social malaise

The majority of women with disabilities did not have a job. This situation made these women a burden on their families and increased their isolation and unhappiness. The example of A.T. illustrates this:

“I would like to have an income-generating activity that can allow me to take care of myself and meet my needs. Sometimes, when I find myself alone, I cry and ask God what sins I have committed or what sins my parents have committed for them to live in all this misery. I would like the institutions that take care of PWD like me to come and get me and put me in a home for PWD to live with people like me because they are the only ones who can understand what I am going through” (A.T., 22 years old, disabled and not in school, Pobè-Benin).

#### 3.2.4 From interventions to the hope of a fulfilling life

The results of univariate and multivariate analyses showed that education, income, occupation and marital status were variables associated with inequity. These characteristics hinder access to healthcare, education, income and social life. We therefore set up interventions to address these issues. the following life stories describe, in sequential order, how these interventions were implemented and contributed to their self-fulfillment. This paper presents three examples of interventions carried out in this context.

##### 3.2.4.1 Mrs. A.L.

At the age of five, A.L., experienced a life-altering event when her foot began to swell, followed by the appearance of a pus. After unsuccessful treatment by a traditional healer and a pastor, her parents finally took her to the Lalo health center, where she was diagnosed with Buruli ulcer. Treatment, which lasted a year, cured her foot, but her father died during this period, complicating her financial arrangements. Later, her second foot was affected, requiring three years of hospitalization. Doctors suggested amputation, but A.L. refused, opting instead for a procedure in which the bones were reinforced with metal. Her disability isolated her socially, making it difficult for her to find friends and companions. She finally found a partner at the age of 25 years (third wife of a polygamous man) and is now expecting her first child but is worried about the prospect of a complicated childbirth. Unable to work, she depends on others to perform daily chores and is considering training as a hairdresser to support herself. Social stigma persists, exacerbated by her mother's mental illness, and A.L. experiences a profound sense of loneliness and isolation.

The economic stability of A.L.'s household depended largely on fluctuations in the price of cereals, which is her husband's main source of income, in addition to farm work. Although her husband takes care of her financially, she sometimes has to call on the help of third parties, whom she pays with the money he gives her. Her husband gives her special attention because of her disability, which requires her to have extra protection. The household has long suffered from stigmatization, but the situation has improved recently as a result of the interventions carried out.

The intervention was implemented gradually, starting with the health component, which included several medical consultations and x-rays of both legs. In terms of livelihood, A.L. benefitted from vocational training. On the social front, the home was rebuilt, and a latrine was installed. In addition to the creation of support groups for PWD by social workers, community awareness was promoted by the Association of Disabled People.

Although A.L. was enthusiastic about the idea of these interventions, she refused the proposed amputation and was equipped with a hearing aid. Her husband also contributed by covering his travel costs for the procedures and providing the sites for the construction of their home and A.L. workshop.

Since starting her apprenticeship, A.L. has gained self-confidence and begun to overcome her shyness. Although her full participation in social life is still a way off, progress is being made. Interventions to support her development are ongoing. Mrs. A.L. is now the mother of one child.

##### 3.2.4.2 Mrs. A.M.

Mrs. A.M. is very introverted. She is the second wife in a polygamous household. She left a marriage in which she was not accepted by the first wife and lost the two children she had. The loss of her children before the age of two years made her very unhappy. She says she suffered an enormous amount of humiliation and frustration.

She became disabled as a result of Buruli ulcer, which she contracted at around the age of seven years. She was hospitalized at the Centre de Traitement et de Dépistage de l'ulcère de Buruli (CDTLUB) in Pobè until the age of twelve years when treatment resulted in the transfemoral amputation of her lower left limb. Her parents were separated, and her mother, who had sole custody of her, struggled to meet A.M.'s basic needs.

Mrs. A.M. benefited from interventions that allowed her to improve her condition. She was awarded a scholarship and enrolled in a boarding school in Pobè, where she completed her primary education and began training in sewing after obtaining her CEP (primary school certificate). She then trained as a seamstress. Following sewing training, she purchased a new machine, which she used for several years until it broke. Since then, her income-generating activities have been limited to knitting and making “KOGUI” soap. The modest income from these activities makes it difficult for her to contribute to household expenses. During her schooling and apprenticeship, mobility was not a significant issue, as the CDTLUB social program provided support and ensured that her school and training centers were located near her home. After her training, her limited mobility restricted her social participation in the Community. In her community, she did not feel rejected or excluded but preferred to remain distant for fear of being ridiculed for her disability. She testified to the constant support of her current husband. Mrs. A.M.'s husband admitted that his activities as a motorcycle taxi driver were temporarily affected to prioritize his travel over greater distances.

To improve their living conditions, A.M. wanted to set up a sewing workshop and purchase a new machine but was unable to do so due to financial limitations. Funding secured through interventions allowed A.M. to build a workshop and shop selling haberdashery and refreshments. She also purchased a new sewing machine and a serger machine. Owing to the effect of awareness-raising by the members of the support group and the social worker on her self-esteem, A.M. ended up eliminating this feeling of self-isolation and managed to join three different associations, namely, the tailors and seamstresses association in her locality and two other women's mutual and credit associations. She gradually became financially independent and was able to save her only living son from premature death, which claimed the lives of her previous sons. She now contributes as best she can to her household's financial expenses. After about a year and a half of activity, her husband told us with a laugh: “She is really strict regarding her accounts and favors her tontines, but I appreciate the fact that she intervenes spontaneously on days when I don't bring anything home”. A.M. is very happy with her current activity. Due to high demand, her haberdashery and refreshment business is very successful. In October 2024, together with the women of her cooperative, she initiated the transformation of cassava into “gari” (cassava flour), an activity that she learned during her sewing training. She was so proud because they sold twelve 100-kilo bags in less than fifteen days.

A.M. is approximately seven months pregnant with her second baby, and everything is going very well with her pregnancy.

##### 3.2.4.3 Mrs. D.D.

D.D. is a girl with a motor disability due to Buruli ulcer in her upper left limb. The fourth of six (6) children, she is originally from Sahouè and currently lives in the house of their church pastor. When she was approximately eight years old, she noticed a small bump resembling an insect bite on her left arm one day after returning from school in Nigeria, where her parents lived. After showing it to her mother, who applied an ointment, the bump grew larger, and her entire arm swelled completely within a week. Her parents sought treatment from a traditional healer and several hospitals in Nigeria, but the treatments were not successful. Therefore, her father decided to bring her back to their village in Benin. Upon arrival, her grandfather referred them to a community health worker in Sahouè, who then referred them to the Buruli Ulcer Screening and Treatment Center in Lalo. There, she underwent the entire course of treatment until she was scheduled for skin graft surgery. She was then taken to CDTUB/Allada by the chief physician, where she received the remainder of her care until her wound healed completely.

She subsequently received a scholarship for her primary education. Following this, her aunt, who was also her guardian, took charge of her studies until she obtained her BEPC (first cycle studies certificate). However, she had to interrupt her studies due to a lack of financial means, and a benefactor then decided to finance her pastry, catering and hospitality training (a training she enjoys) until she obtained her Certificate of Professional Aptitude (CAP). Despite obtaining this certificate, she faced difficulties in finding stable employment due to her disability. She said, “I was not able to continue my training as I wanted due to lack of means”. During her training, she also experienced stigma from her peers, which affected her deeply and led her to isolate herself further. The economic situation of the household was precarious. Only her mother, who resides in Nigeria, managed to provide for her occasionally, but this was no longer possible owing to the fluctuation of the naira. D.D. was not receiving any other support, and this intervention came at the right time, giving her hope of becoming independent. Her aunt, who has grown old, is no longer able to provide for herself or her niece. D.D.'s wish was to complete her training and obtain the State Technical Diploma, but owing to a lack of means, she had to stay at home and could not even perform professional internships because of the constant rejections she experienced. She was very happy to benefit from this support. The support she received had a positive impact on multiple areas of her life, including health, livelihood, education, and social connections. As a result of the intervention, she received a full hospitality training scholarship, training in self-esteem and lifeline management, and joined the Association of Disabled People linked to MTN-mc of Benin.

This intervention also allowed her to build an equipped mobile pastry shop. Awareness-raising was carried out in collaboration with the administration of her school to ensure her effective reintegration. She is no longer discriminated against at her school. Owing to training in self-esteem and personal development, she is better integrated into her environment and no longer stigmatizes herself.

## 4 Discussion

The existing literature examining the intersectionality between gender and disability remains limited, particularly in African countries such as Benin and Côte d'Ivoire. While there is growing recognition of the need to explore how gender and disability intersect to shape the experiences of individuals, most studies have focused on either gender or disability independently, often overlooking the nuanced and compounded challenges faced by people at the intersection of both. This study showed that women with disabilities face difficulties in their marital and professional lives.

The study population was young, with a median age of 38 years and a predominance of males (sex ratio = 1.45). The young population is simply a reflection of the general population of these localities in Benin and Côte d'Ivoire. Average life expectancy is around 57.8 years and the median age is 17.9 years in the WAEMU (West African Economic and Monetary Union) area (27). The predominance of men could be explained by the fact that they do not need the approval of their partners to take part in the study, unlike women, who may not have received the agreement or authorization of their partners or who do not have access to care at treatment centers. Moreover, NTDs are more commonly detected in men than in women, considering that women are more isolated, rejected, and stigmatized (28). Uneducated status exposes women to domestic violence, which in turn is encouraged by unemployment, which creates financial dependence on the partner (8, 29, 30). In our study, 42.8% of women had not attended school, and 49.2% of women are unemployed.

This study revealed that over half of the people with disabilities (PWD) had not attended school, indicating a significant lack of educational access. This observation is consistent with the documented stigma often faced by PWD. While Benin and Côte d'Ivoire have achieved comparatively higher schooling levels than some other sub-Saharan African countries (as highlighted by Mvodo Victor Stéphane's work), inequities persist.

This study aligns with the perspective of differential schooling based on gender, a trend also observed in Cameroon. Data from Cameroon's 2005 general population census, for example, showed a net enrollment rate of 69.9% for young people with disabilities, with higher enrollment among boys than girls in both urban and rural areas. These enrollment rates remain below the national average, further demonstrating gender-based inequities (31).

Mbom concluded that nonattendance rates for disabled children are higher in male-headed households than in female-headed households (32). A different finding was reported in Sharon Eva A's study conducted in 2014, in which women were more highly educated than men (33). This difference could be explained by the relatively small sample size compared with that of the present study. According to Charles Emmanuel Mouté Nyokon, PWD are less likely to be in school (78% vs. 100% for men and 80% vs. 100% for women, *p* < 0. 001 for both sexes) than people without disabilities, with more than half of disabled participants having a primary school education or less (61% vs. 22% for men and 57% vs. 33% for women; *p* < 0.001 for both sexes) (34). Similarly, a study on gender inequities among disabled children in Cameroon revealed that boys' school attendance rates are higher than those of girls (32).

Mac-Seing M argues that girls' under-schooling is influenced by several factors, including sociocultural constraints, parental education levels, and the characteristics of the head of household (especially their gender). Household standards of living and composition, particularly the number of children under five, are also key factors (35).

Married people predominated (43.7%), followed by single people (35.6%). There were more married men than married women. The significant association between gender and marital status (*p* = 0.000) indicates that men and women with disabilities have very different marital experiences. In the African context, cultural and social norms assign distinct gender roles to men and women, and this is reflected in the way PWD are perceived in the context of marriage. A study conducted in Africa showed that there is a complex interplay between traditional values and new gender perspectives, influenced by factors such as globalization, education and activism. It was shown that, despite the progress made, the persistence of prejudice and gender inequality highlights the incomplete nature of this transformation (36).

Furthermore, a study of the marital status of disabled women in Canada suggested that they are less likely to be married or in a cohabiting relationship (37). Among disabled women, those with early onset conditions, cognitive impairment, mobility limitations and lower levels It of educational attainment are more likely to remain single, that is, never having entered into a cohabiting relationship. Disabled women have less opportunity to meet potential partners and form lasting cohabiting relationships (37). Acharya et al. concluded that girls with disabilities had more difficulty getting married than boys (38). Disability can lead to self-stigmatization, which means that people with disabilities prefer to isolate themselves for fear of how others will look at them, as stated by Madams AL, AM and DD, who were self-isolated and self-stigmatized. Nosek et al.'s work with disabled women concluded that: disabled women had significantly lower self-knowledge and self-esteem and greater social isolation than non-disabled women, as well as significantly lower levels of education, greater overprotection during childhood, poorer quality intimate relationships, and lower rates of paid employment (39). Awareness-raising and training activities for the husbands and family members of PWD could help restore confidence in PWD and promote inclusive social transformation.

Indeed, disabled men are much more tolerated and accepted as spouses and fathers than their female counterparts, who are often rejected and not accepted as having the right to a family life or even to have children. The fear is that the children of disabled women will also carry the same defects or curses. Disabled women also face increased discrimination regarding to marriage, being perceived as less “desirable” because of stereotypes linked to their disability and their ability to take on domestic and family responsibilities. While facing barriers, men may be perceived as providers and may therefore have a greater likelihood of marrying despite their disability (38, 39).

Women with disability are more likely to be shopkeepers/housekeepers, for example, than managers, craftsmen, or farmers. In terms of occupation, almost three-quarters of the PWD had no occupation at all. The relationship between gender and occupation type (*p* = 0.000) shows that gender strongly influences the type of jobs to which PWD have access. Women with disabilities, in particular, are often confined to informal or low-skilled jobs because their low level of education and cultural expectations limit their active participation in economic life. On the other hand, men with disabilities may have more opportunities to access more stable or better-paid jobs, even if their situation remains precarious. This finding highlights the need for specific education and professional programs for women with disabilities to enable them to better integrate into the labor market and gain access to valued professions.

These findings are in line with those of Jessiman-Perreault G et al. in a 2024 cross-sectional survey of 2307 Canadian workers titled “Understanding the Unmet Accommodation Needs of People Working with Mental or Cognitive Conditions: The Importance of Gender, Gendered Work, and Employment Factors”, who noted that gender may be an important factor, as women are more likely to have precarious working conditions and are more likely to experience job insecurity than men are. Women report a greater number of adaptation needs than men do (40). According to the same author, an earlier study revealed that women with and without disabilities are more likely to have unmet support needs at work than men without disabilities are (40).

The interactions among sex, gender, and disability in the context of working conditions needs to be examined in greater detail. To better understand the role of gender in work and health, it may be important to consider differences in jobs and work tasks, particularly when considering the segregation of men and women that traditionally exists in certain professions. Glauber reported that gender-neutral occupations offer more opportunities for flexible working than do either male-dominated or female-dominated occupations. However, studies have not explored the potential intersectional effect of being a person with a disabling condition and being employed in occupations with a different gendered distribution (i.e., gender-neutral, female-dominated, and male-dominated occupations) (40).

This may suggest that there is something unique about the availability of workplace accommodations and support in jobs occupied by women that increases the risk of unmet requirements beyond the working conditions and contexts examined in this study. For example, women are overrepresented in jobs that tend to be more customer- or service-oriented, and it may therefore be more difficult to allow flexibility in work location or hours for individuals with these types of jobs. In addition, women are more likely to have positions characterized by precarious work factors, which may make them hesitant to request accommodations due to the risk of reprisal (40). The characteristics of the first job held are significantly different between men and women with disabilities but not between men and women without disabilities (41). Most PWD have informal employment, with women predominating (56% of men and 64% of women), whereas most people without disabilities have formal employment (42).

In contrast to our results, those of Mac-Seing M et al. revealed that there was no difference between disability groups in the effect of gender identity or occupational gender distribution on the log scale for any of the outcomes. They noted that jobs associated with contract work, part-time hours and precarious working conditions, such as less control over employment, greater job insecurity, more precarious wages and greater vulnerability, are more likely to be occupied by people with mental or cognitive disorders leading to disability and by women (35).

More than three-quarters of the respondents (89.2%) had a monthly income between 0 and 76 euros. The association between gender and monthly income (*p* = 0.000) reveals a significant disparity in the economic opportunities of disabled men and women. In many sub-Saharan African countries, women's access to economic resources is often limited by lower educational attainment, labor market discrimination, and cultural expectations surrounding domestic roles. For women with disabilities, these barriers are often exacerbated, severely limiting their access to well-paid jobs or income-generating activities. These results demonstrate the importance of targeted public policies to improve disabled women's access to economic opportunities and reduce the gender income gap.

The fact that engaging in an income-generating activity was significantly associated with gender (*p* = 0.000) reflects the same dynamics observed in the other factors. Disabled women are often at a disadvantage in accessing the resources they need to start or maintain an income-generating activity because of the multiple types of discrimination they experience, both as women and as disabled people. This result shows that the economic empowerment of people with disabilities, especially women must be *a priori*ty in development programs in sub-Saharan Africa to promote their financial independence and active participation in social and economic activities.

A study by Olena Stryzhak has shown that education is closely linked to income level and awareness of happiness (43). In our study, the majority of disabled people (regardless of gender) were unschooled, had no occupation and earned between 0 and 76 euros a month. Intervention in these areas (as was the case for Ms A.L., A.M. and DD) could enable PWDs to achieve greater self-fulfillment.

According to Charles Emmanuel Mouté Nyokon's study in Cameroon, individuals with disabilities are significantly more likely to live in poor households compared to those without disabilities. Specifically, 30% of men with disabilities lived in poor households compared to 19% of men without disabilities (*p* = 0.006, odds ratio = 1.58). Among women, 28% with disabilities lived in poor households compared to 19% of those without disabilities (*p* = 0.021, odds ratio = 1.47). These results indicate a statistically significant association between disability and poverty in Cameroon (41).

When interpreting our results, it is important to nuance and take into account that some of the independent variables such as education, income, occupation and marital status used in our study could be confounding factors For example, education and income can be related regardless of gender issues. Indeed, education level could influence income level, as generally higher-educated people are likely to have higher incomes and therefore be subject to less discrimination and inequality. Thus, individuals with different levels of education but the same income will not have different levels of discrimination. These observations suggest that the data need to be looked at differently, considering the potential interactions between the variables.

## 5 Limitations

This study has some limitations. Individuals with disabilities and severely deteriorated health may have been absent from their place of residence or community during data collection. Reliance on self-reported data may have introduced recall bias. While rigorous interviewer training and pre-testing of translated questionnaires aimed to minimize information bias associated with simultaneous translation, the potential for such bias remains.

## 6 Conclusion

Employing an intersectional lens, this study offers a culturally sensitive analysis of the complex inequities experienced by women and girls with disabilities in Benin and Côte d'Ivoire. By focusing on the intersection of disability and gender, the research reveals distinct challenges that differ from those faced by men with disabilities and women without disabilities. The study concludes that effective interventions must address the specific medical, social, and economic needs of women with disabilities at the individual, household, and community levels to achieve meaningful change. The gender inequities identified in this study have direct implications for public health. PWD, particularly women, are more likely to live in poverty and have limited access to health services, which exacerbates their vulnerability. These findings underscore the need for an inclusive, gender-based approach to public health policymaking in Benin and Côte d'Ivoire and, by extension, throughout the WAEMU region to ensure that women with disabilities benefit from equitable access to healthcare, education and economic opportunities.

To achieve social equality and equity for PWD in sub-Saharan Africa, public policies, aid programs, and development initiatives must consider existing gender disparities. This study calls for the integration of gender and disability considerations into all aspects of policy development, service provision, and community-based initiatives. This includes not only the protection of women with disabilities from violence and discrimination but also their active participation in decision-making processes, ensuring that they have a voice in shaping policies that affect their lives.

## Data Availability

The raw data supporting the conclusions of this article will be made available by the authors, without undue reservation.
